# The Generalizability of Patients' Preferences and Concerns regarding Anesthesia Care for Cesarean Delivery: A Prospective Survey

**DOI:** 10.1155/2021/9002061

**Published:** 2021-12-02

**Authors:** Aaron J. Smith, Jaime Daly, David E. Arnolds, Barbara M. Scavone, Brendan Carvalho

**Affiliations:** ^1^Departments of Anesthesia and Critical Care, The University of Chicago, 5841 S. Maryland Ave, Chicago, IL 60637, USA; ^2^Departments of Anesthesia and Critical Care and Obstetrics and Gynecology, The University of Chicago, 5841 S. Maryland Ave, Chicago, IL 60637, USA; ^3^Department of Anesthesia, Stanford University, 300 Pasteur Dr., Stanford, CA 94305, USA

## Abstract

**Background:**

False assumptions regarding the generalizability of patients' expectations and preferences across different demographic groups may contribute in part to the increased prevalence of negative peripartum outcomes seen among women of color. The intention of this study was to determine preferences and concerns regarding anesthesia care during cesarean delivery in a largely African-American population and to compare them to those obtained in a prior study conducted in a demographically distinct population.

**Methods:**

Women presenting for scheduled cesarean delivery or induction of labor completed a preoperative survey requesting demographic information and the opportunity to rank ten common potential anesthetic outcomes in relation to each other from most to least desirable. Participants were also asked about their biggest fear concerning their anesthetic and their preferences and expectations regarding degree of wakefulness, pain, and other adverse events. Those who underwent cesarean delivery were administered a briefer postoperative survey. We tabulated preference rankings and then compared demographic and outcome data to that obtained in a previous study with a demographically dissimilar population.

**Results:**

A total of 73 women completed the preoperative survey, and 64 took the postoperative survey. Pain during and after cesarean delivery was ranked as least desirable outcomes and fear of paralysis was respondents' principal concern with neuraxial anesthesia. Postoperative concerns were similar to preoperative concerns and did not correlate with the frequency with which specific adverse outcomes occurred. These results were consistent with those from the previous study despite the women in this study being more likely to be younger, unmarried, African-American, and less educated than those in the previous investigation.

**Conclusions:**

Patient preference rankings and concerns were remarkably similar to those previously demonstrated despite a number of demographic differences between the two populations, suggesting generalizability of these preferences to a broader obstetric population.

## 1. Introduction

Medical care in the United States has evolved from emphasizing physician paternalism to stressing patient autonomy. Although surgical patients' anesthetic expectations and outcome preferences have been documented [1], less is known about obstetric patients' expectations and preferences for anesthesia outcomes surrounding cesarean delivery. Patients' concerns for pain and associated side effects impact their preferences for analgesics after cesarean delivery [2, 3]. Greater understanding may allow clinicians to better tailor anesthetic plans to improve both patient satisfaction and quality of medical care.

Researchers at Stanford University developed and administered a standardized survey to study patient preferences for anesthetic outcomes after cesarean delivery [4]. The survey determined the preference ranking of potential anesthetic and related side effects as well as subjects' greatest fear of neuraxial anesthesia [4]. The authors determined that avoiding both intraoperative and postoperative pain represented patients' greatest concern during the perioperative period and that paralysis represented their most prominent fear of neuraxial anesthesia. However, most subjects in that study were Caucasian, had post-bachelor degree education, and earned median annual household incomes between $50,000 and $100,000. Therefore, the ability to generalize and extrapolate the findings to different obstetric populations and settings may be limited as different preferences and concerns may exist among disparate ethnic and socioeconomic groups. It is important to examine racial and ethnical differences in inclinations, expectations, and apprehensions regarding anesthetic outcomes after cesarean delivery. Determining the generalizability of patients' preferences and concerns is important because false assumptions may lead to communication errors, negatively impact care, and contribute in part to the increased prevalence of negative outcomes among women of color [5].

In this study, we closely followed the previous survey methodology utilized by the Stanford group [4] and applied it to a predominately African-American population with fewer years of education, and who are from an area of Chicago where many residents have limited financial means. The primary aim of the study was to determine preference rankings of potential anesthesia outcomes in this patient population. A secondary aim was to compare pre- and postdelivery rankings. We also compared our results to those of the previous study cohort [4]. We hypothesized that preferences and concerns regarding anesthetic outcomes and potential side effects after cesarean delivery in our patient population might differ from those determined in the Stanford study.

## 2. Materials and Methods

This study was approved by the University of Chicago Institutional Review Board (no. 16–1767, approved December 26, 2016). We approached pregnant women presenting for either scheduled cesarean delivery or scheduled induction of labor at The University of Chicago Medicine (UCM) Hospital from July 2017 to June 2019 to participate in this study. Written informed consent was obtained by a study investigator immediately following the routine preanesthesia interview on the day of admission. Women presenting in painful labor, defined as a pain score ≥3/10, were excluded from participation.

Consenting subjects were then administered a survey adapted from the one developed by the Stanford group [4] In addition to providing detailed written instructions, the study investigator verbally explained the various tasks in the survey to improve the participants' understanding. The investigator remained present throughout the entire survey administration, verbally reading each survey question, answering questions about the survey questions, and allowing the patient to record her answers with pen and paper. The survey (Appendix) contained questions about demographic variables including age, marital status, self-identified race, maximum educational level attained, and annual household income. Each woman was also asked whether or not she had taken formal prenatal classes or was a healthcare professional and about previous experiences with surgery and anesthesia.

Perioperative expectations and preferences were assessed by (1) asking survey respondents to rank ten common potential anesthetic and related side effect outcomes in relation to each other from the most desirable [1] to the least desirable [6] and (2) requesting women to distribute a hypothetical $100 among the same ten potential anesthetic outcomes in order to prevent undesirable outcomes such that the more money spent on a condition, the less likely it would occur. Initially subjects performed both the ranking and the money distribution exercises to assess their preferences regarding anesthetic outcomes. However, part way through the study, we began administering only the first exercise (ranking 1 through 10) and not the second one (distributing $100) because the distribution of money seemed to cause confusion among some participants, and the Stanford group did not detect a difference between these two relative value measurement techniques [4].

Participants were asked about their preferred degree of wakefulness during surgery, their expectations of pain, how much pain (on a visual analog scale 0–10 cm scale, 0 cm = no pain, and 10 cm = worse pain) they would tolerate rather than expose their baby to medication, and their biggest fear of neuraxial anesthesia (from a list of several choices). Women also completed a “trade-off” table forcing them to make choices between pain relief and several common opioid treatment-related adverse effects (nausea, vomiting, and pruritus). This “trade-off” table allowed investigators to determine how women balance potential benefits and side effects of postoperative analgesia.

All patients who delivered via cesarean delivery (both those patients who had scheduled cesarean delivery and those patients admitted for induction of labor who underwent unplanned cesarean delivery) were asked to fill out a briefer survey on postoperative day 1 during the routine postanesthesia visit. The postdelivery survey included the same priority ranking, money distribution exercise (later eliminated), and “trade-off” table as the predelivery survey.

### 2.1. Statistical Analysis

The primary outcome variable of interest was the priority ranking for potential anesthesia and related side effect outcomes in the study cohort. A secondary outcome variable was to compare preoperative to postoperative priority rankings. We also compared preferences in the current survey conducted at the UCM to the Stanford study conducted at Lucile Packard Children's Hospital (LPCH). We obtained the raw data from the Stanford group in order to allow for the between-institution comparisons. Since the primary outcome was descriptive in nature, we did not perform a sample size or power analysis but rather chose a convenience sample similar to that used by the Stanford group (*n* = 82) [4].

Data are presented as mean ± standard deviation, median (interquartile range), and number (percentage, %). Categorical data were compared between groups with Chi-squared or Fisher's exact test as appropriate, and continuous data were compared between groups with Student's *t*-test or Mann–Whitney *U* test as appropriate. In order to compare preoperative to postoperative ranking, we first dichotomized ranks as low-rank (rank ≤5) and high-rank (rank >5). Then low versus high rank between pre- and postoperative anesthetic and related side effect outcome preferences per category was analyzed using McNemar's test. The between-institution priority ranking analysis was simplified to identify and compare those patients who indicated pain either during or after surgery as the least desirable outcome. We also identified and compared those patients whose biggest fear was paralysis. A two-sided *P* < 0.05 was required to reject the null hypothesis. We used STATA version 15.1 for all analyses.

## 3. Results

We administered 74 preoperative surveys, one of which was incomplete and excluded from analysis, leaving a total of 73 preoperative surveys. A total of 70 of these 73 subjects underwent cesarean delivery, and 64 of them completed a postoperative survey (5 declined to participate and 2 were discharged before completing it).

Demographic information of the study population is listed in Table 1. In comparison to the LPCH population, our subjects were younger, less likely to be married, more likely to identify as African-American, and received less formal education. There was also a nonsignificant trend toward lower household income. The UCM population was more likely to be multiparous and had experienced both a prior cesarean delivery and spinal or epidural anesthesia. Seven subjects (10%) reported taking prenatal classes, compared to 100% of those in the Stanford population (*P* < 0.0001). There was no difference in representation of healthcare workers or women with a history of previous (noncesarean delivery) surgery requiring anesthesia.

Pre- and postdelivery priority ranking of potential anesthetic outcomes are outlined in Table 2, as is the frequency of unpleasant outcomes experienced postdelivery. We found no difference in pre- and postdelivery ranking order. On the day after surgery, 85% of patients reported experiencing at least one unpleasant side effect during or after their cesarean delivery. Itching was the most commonly reported side effect, followed by nausea, vomiting, and nervousness before surgery. When asked “What was more painful, the IV or spinal/epidural placement?” 73% of patients selected “spinal/epidural placement.” When asked, once again, to select a preferred anesthetic dose from the “trade-off” table, 63% of women preferred a “moderate dose,” 34% selected “highest dose,” and 3% chose “lowest dose.”

Predelilvery survey responses regarding preferences and expectations of anesthesia for cesarean delivery are listed in Table 3. No differences were found regarding preferences and expectations surrounding cesarean delivery between the UCM and LPCH populations. In both populations, the majority of women preferred to be awake during surgery. Both groups most commonly designated “pain during” or “pain after” surgery as the least desirable outcome. When asked “What is your biggest fear?” concerning their anesthetic, a relatively large proportion of respondents in both groups selected “paralysis.” Response choices in the UCM population included paralysis (42 (59%)), backache (9 (12%)), pain on insertion (8 (11%)), failure to provide pain relief (6 (8%)), headache (1 (1%)), and other (4 (5%)). A sizable minority in both groups expected no postoperative pain. Women in both groups anticipated tolerating a median visual analog pain score of >5 cm (out of 10 cm) rather than exposing their baby to analgesic medication.

## 4. Discussion

Patient preferences and concerns regarding potential anesthesia outcomes and related side effects in the current patient population were remarkably similar to those demonstrated in the previous Stanford study cohort [4]. These similarities were apparent despite a number of demographic differences between the study populations. Specifically, women in the current investigation were more likely to be younger, unmarried, African-American, receive less formal education, and demonstrate a trend toward lower household income. The UCM study population was also more likely to be multiparous and has experienced both a prior cesarean delivery and neuraxial anesthetic. Despite these differences in demographics and obstetric characteristics, both groups of women ranked preference for potential anesthetic and associated side effect outcomes in identical fashion (in order from least to most desirable: pain during surgery, pain after surgery, vomiting, nausea, cramping, itching, shivering, nervousness, and sleepiness). The desires to avoid pain, minimize fetal analgesia exposure, and remain awake during the survey respondent's cesarean delivery were also very similar in both of these study cohorts. These results suggest that pregnant women's preferences for anesthesia outcomes are likely universal and generalizable to a broader obstetric community and appear not to be significantly influenced by socioeconomic factors such as age, race, marital status and education level, or obstetric factors, such as parity or previous cesarean delivery. Neither do they appear to be influenced by formal prenatal education. All of the Stanford patients underwent such training—the study having been conducted during prenatal classes—compared to only 10% of the UCM patients. This may indicate a need for inclusion of more specific information relating to anesthesia in prenatal curricula.

Women ranked pain during and after surgery as their leading concerns, and ahead of potential anesthesia-related side effects including vomiting, nausea, cramping, itching, and shivering. Undergoing cesarean delivery did not significantly influence concerns regarding anesthesia and side effect outcomes; preferences reported in the postoperative survey were nearly identical to those in the preoperative survey. The priority ranking for potential anesthesia and associated side effect outcomes postdelivery was unchanged despite variation in the frequency of outcomes actually experienced. For instance, 18% of patients reported experiencing pain after surgery, and nearly half reported nausea and vomiting; however, the overall ranking still found pain ranked higher than nausea.

Several investigators have identified pain as the least desirable outcome among nonobstetric general surgery patients [7–10]. Other studies, however, have revealed postoperative nausea and vomiting as more concerning to surgical patients than pain [1, 6, 11]. Our finding that women at both UCM and Stanford ranked “pain during surgery” and “pain after surgery” as less desirable than nausea/vomiting suggests that avoidance of pain is a greater priority for obstetric versus general surgical patients. We hypothesize that this may be due to cesarean delivery being performed in awake women with neuraxial anesthesia and that many pregnant women experience nausea and vomiting during pregnancy and may therefore be less concerned about nausea and other side effects.

Unique considerations among obstetric patients such as exposing the fetus to medication and being able to breastfeed and care for a newborn shortly after surgery are reflected in preferences for analgesia dose and the degree of pain one would tolerate before requesting analgesia. We found, consistent with the Stanford study, that women believed they would tolerate moderate pain rather than expose their baby to medication. These results highlight that anesthesia providers should elicit patients' understanding of the degree of opioid transmission both transplacentally and via breastfeeding during the preoperative assessment, and should educate patients regarding the safety of postoperative analgesics, as this potential exposure appears to be a concern for women. Future studies investigating patients' understanding of analgesic transmissibility to newborns would help inform discussions about risks and benefits.

When offered a hypothetical “trade-off” between pain relief and side effects, the majority of patients would rather have a “moderate dose” of pain medication to offset potential side effects. These findings were similar to those reported in the Stanford cohort [4]. Women's preference for selecting the “moderate dose” was also investigated in a prospective patient-choice study in which approximately two-thirds of women selected the middle analgesic dose [3]. Interestingly, women have insight into their analgesic needs in that those who chose the higher dose analgesic protocol had greater postoperative opioid requirements, and those who chose the lower dose analgesic protocol required fewer opioids after their cesarean delivery [2, 3]. Furthermore, selection of the high dose is driven by concerns related to pain, and selection of the lower doses is driven by aversion to side effects [2, 3]. Our survey results, coupled with these patient-choice studies, suggest that these fears and concerns for potential anesthesia outcomes and related side effects should be addressed in a patient-engaged preoperative anesthesia interview.

Although the incidence of neuraxial anesthetic-related paralysis in obstetric patients is exceedingly low [12–14], paralysis was, surprisingly, the most common “biggest fear” among respondents. These results are in line with previous regional anesthesia studies that found patients are disproportionately fearful of rare adverse outcomes [11, 15], suggesting that patient education regarding risks of anesthetic procedures may be inadequate. Epidural side effects and risks are among the most common birth-related epidural Google searches [16]. Anesthesia providers should reassure patients that severe adverse neurologic events related to neuraxial anesthesia are extraordinarily rare, especially in an obstetric setting [14, 17, 18]. Additionally, anesthesia providers should emphasize the preventative measures that will be used and the monitoring that will be undertaken during the anesthetic to further reduce fear and anxiety.

This study has several strengths. Our high postoperative survey completion rate allowed us to compare preoperative perceptions of side effect outcomes with the actual frequency of outcomes experienced. Also, we were able to demonstrate several demographic differences between subjects in our population versus the Stanford cohort [4], allowing us to draw the conclusion that patient preferences and concerns surrounding cesarean delivery and anesthesia transcend many several socioeconomic and obstetric differences. The authors of both manuscripts were in direct communication throughout the study's design and execution, and the study methodology used in the Stanford study was followed as closely as possible in order to effectively compare the two populations.

The study also has potential limitations. Despite having a research team member present and available for questions during survey administration, many patients had difficulty understanding and accurately responding to certain questions. The dollar allocation section in particular caused significant confusion for many patients and was ultimately excluded from analysis. Interestingly, the Stanford group did not encounter similar issues despite using identical questions. In the present investigation, surveys were administered in the preoperative area shortly before patients anticipated having surgery, whereas the Stanford investigators conducted the survey at expecting parent class weeks before the delivery. Perhaps administering the survey in a more comfortable setting where patients have more time to respond leads to better comprehension of survey questions. Also, the Stanford group asked subjects to mail in responses whereas we conducted interviews in person. Furthermore, the Stanford study was conducted several years ago in 2002–2004. Any of these differences could have impacted between-institution comparisons, but the fact that both groups ranked outcomes in precisely the same preference order lends credibility to our finding that there were no differences in outcome variables between the two groups. Lastly, the multiple-choice format of the question “What is your biggest fear regarding spinal/epidural anesthesia?” most likely led many patients to select the most frightening option, “paralysis.” A free response format might prove more informative of patients' preexisting fears and knowledge of potential adverse outcomes. Furthermore, including risk quantification that appropriately identified paralysis as rare might have allayed patients' concerns about this outcome.

## 5. Conclusions

Results of this study add to our limited knowledge of obstetric patients' preferences and concerns regarding anesthesia outcomes and related side effects for cesarean delivery. They suggest that obstetric patients' preferences and concerns are fairly uniform and not influenced by racial, ethnic, and socioeconomic differences. Our results highlight the importance of adequate discussion of risks and benefits between providers and patients of all demographic backgrounds and the importance of eliciting individual patient's concerns and considering them when designing anesthetic plans.

## Figures and Tables

**Table 1:** Demographic characteristics of respondents from the University of Chicago Medicine compared to those from the previous Lucile Packard Children's Hospital, Stanford study^*∗*^.

		UCM *n* = 73	LPCH *n* = 82	*P* value
Age (years)		30 ± 6	33 ± 4	0.0001

Marital status	Married	29 (40)	76 (93)	<0.0001
	Unmarried	44 (60)	5 (7)	

Ethnicity	African-American	49 (67)	1 (1)	<0.0001
	Hispanic	12 (16)	13 (16)	
	Caucasian	7 (10)	58 (71)	
	Others	5 (7)	8 (10)	

Years of schooling	Some college/high school (<16 yrs)	51 (70)	11 (13)	<0.0001
	Bachelor's degree (16 yrs)	11 (15)	26 (32)	
	Graduate degree (>16 yrs)	11 (15)	45 (55)	

Household income	<$20,000	22 (30)	0 (0)	0.33
	$20–50,000	25 (34)	3 (4)	
	$50–100,000	15 (21)	15 (18)	
	$100–200,000	8 (11)	39 (48)	
	>$200,000	3 (4)	21 (26)	

Healthcare professional		9 [12]	10 (12)	1.0
Previous surgery requiring anesthesia		52 (71)	63 (77)	0.54
Previous cesarean delivery		50 (68)	15 (18)	<0.0001
Previous epidural or spinal anesthesia		41 (56)	21 (26)	0.0002

UCM: University of Chicago Medicine; LPCH: Lucile Packard Children's Hospital, Stanford. Data presented as mean ± standard deviation or number (percentage). ^*∗*^Carvalho B, Cohen SE, Lipman SS, Fuller A, Mathusamy AD, and Macario A. Patient preferences for anesthesia outcomes associated with cesarean delivery. *Anesth Analg.* 2005; 101 (4): 1182–1187.

**Table 2:** Priority rankings for potential anesthesia-related side effects and their actual frequency.

Outcome	Predelivery rank	Postdelivery rank	*P* value	Frequency experienced
Pain during surgery	9 (8–10)	9 (8–10)	1.0	2 (3)
Pain after surgery	9 (5–10)	9 (4–10)	1.0	11 (18)
Vomiting	7 (5–8)	7 (5–8)	0.15	30 (48)
Nausea	6 (5–8)	7 (6–8)	0.33	30 (48)
Cramping	6 (5–8)	6 (5–7)	0.29	0
Itching	5 (4–7)	5 (4–7)	1.0	38 (61)
Shivering	5 (4–6)	5 (4–6)	1.0	9 (15)
Nervousness	4 (3–5)	3 (3–6)	0.09	17 (27)
Sleepy	3 (2–4)	2 (2–4)	1.0	0
Good recovery	1 (1–2)	1 (1–1)	1.0	N/A

Data presented as median (IQR) or number (percentage). Participants ranked outcomes from the most desirable (1) to the least desirable (10). Six surveys that contained invalid ranking data (e.g., recording “10” in more than one category) were excluded from analysis.

**Table 3:** Cesarean anesthesia preferences and expectations from the current University of Chicago Medicine survey compared to those from the previous Lucile Packard Children's Hospital, Stanford study^*∗*^.

		UCM	LPCH	*P* value
Preferred wakefulness during cesarean	Asleep	9 (12)	13 (16)	0.53
	Awake but sleepy	15 (21)	21 (26)	
	Awake	49 (67)	47 (58)	

Biggest fear is “paralysis”		42 (59)	40 (49)	0.26

Least desirable outcome is “pain during” or “pain after” surgery		51 (76)	50 (70)	0.57

Preferred anesthetic dose	Lowest	7 (10)	4 (8)	0.75
	Moderate	40 (55)	30 (60)	
	Highest	25 (35)	16 (32)	

Expected pain after surgery	None	15 (21)	11 (14)	0.28
	Some	38 (52)	50 (62)	
	Moderate	18 (25)	18 (22)	
	Severe	2 (3)	2 (2)	

Predicted pain tolerance		6.3 ± 2.4	5.7 ± 2.2	0.08

UCM: University of Chicago Medicine; LPCH: Lucile Packard Children's Hospital, Stanford. Data presented as number (percentage) or mean ± standard deviation. Because some patients did not answer every question, percentages are reported as proportion of total responses for each question. ^*∗*^Carvalho B, Cohen SE, Lipman SS, Fuller A, Mathusamy AD, and Macario A. Patient preferences for anesthesia outcomes associated with cesarean delivery. *Anesth Analg.* 2005; 101 (4): 1182-11.

**Table 4:** Surgical history.

Previous surgery (type)	Anesthetic (circle)	Complications (circle)
	GA/regional/MAC or local	Nervous before surgery
		Nausea
		Vomiting
		Pain during surgery
		Pain after surgery
		Shivering during surgery
		Itching
		Others (specify):

	GA/regional/MAC or local	Nervous before surgery
		Nausea
		Vomiting
		Pain during surgery
		Pain after surgery
		Shivering during surgery
		Itching
		Other (specify):

	GA/regional/MAC or local	Nervous before surgery
		Nausea
		Vomiting
		Pain during surgery
		Pain after surgery
		Shivering during surgery
		Itching
		Other (specify):

	GA/regional/MAC or local	Nervous before surgery
		Nausea
		Vomiting
		Pain during surgery
		Pain after surgery
		Shivering during surgery
		Itching
		Others (specify):

GA=general anesthesia; MAC=monitored anesthetic care.

**Table 5:** Obstetric history.

Previous cesarean	Anesthetic (circle)	Complications (see list)
1	GA/neuraxial	Nervous before surgery
		Nausea
		Vomiting
		Pain during surgery
		Pain after surgery
		Shivering during surgery
		Itching
		Others (specify):

2	GA/neuraxial	Nervous before surgery
		Nausea
		Vomiting
		Pain during surgery
		Pain after surgery
		Shivering during surgery
		Itching
		Others (specify):

3	GA/neuraxial	Nervous before surgery
		Nausea
		Vomiting
		Pain during surgery
		Pain after surgery
		Shivering during surgery
		Itching
		Others (specify):

4	GA/neuraxial	Nervous before surgery
		Nausea
		Vomiting
		Pain during surgery
		Pain after surgery
		Shivering during surgery
		Itching
		Others (specify):

5	GA/neuraxial	Nervous before surgery
		Nausea
		Vomiting
		Pain during surgery
		Pain after surgery
		Shivering during surgery
		Itching
		Others (specify):

GA=general anesthesia; MAC=monitored anesthetic care.

**Table 6:** Dollars allocation.

Your rank	Dollars allocation	Description of possible anesthesia outcomes
________	________	Pain: During surgery, you feel some pain and discomfort while the surgeon is doing the cesarean section.
________	________	Itching: You are itching all over your body. Scratching does not relieve the itching.
________	________	Nausea: You want to throw up, but cannot. Your mouth is dry and you are sweating. You feel worse if you move.
________	________	Nervousness: You are scared and anxious about the surgery.
		You are jittery like the feeling you get prior to speaking in public.
________	________	Shivering: Your large muscles start to shake and the shivering travels to your whole body that shakes uncontrollably.
________	________	Cramping: You have intermittent abdominal and uterine cramps. You experience waves of spasm and painful cramps.
________	________	Good recovery: You are lying comfortably in the recovery room. You have no pain and no nausea, feel good, and are able to spend time with your baby.
________	________	Sleepy: During and after surgery you feel tired and unable to remain awake, drifting off to sleep continuously.
________	________	Vomiting: You want to retch. You cannot eat or keep any liquids down.
________	________	Pain: After surgery, your surgical incision really hurts.
		Movement makes the pain worse and no position makes the pain better.

**Table 7:** Medication choice.

Possible side effects	Pain medication dose		
	Lowest dose	Moderate dose	Highest dose
Risk of pain	↑↑	↑	0
Risk of nausea/vomiting	0	↑	↑
Risk of itching	↑	↑	↑↑

## Data Availability

Readers can access the data by emailing bscavone@dacc.uchicago.edu.

## Appendix

### (A). Patient Expectations and Preferences Regarding Anesthesia Care Surrounding Cesarean Delivery Preoperative Survey

Subject # _____

Modern anesthesia care is very safe. However, side effects can occur. Sometimes, your anesthesia doctor must make treatment decisions which “trade-off” an increased risk of one side effect (e.g. a chance of nausea) in order to decrease the risk of pain after cesarean section. The purpose of this survey is to determine your preferences about various possible outcomes of surgery and anesthesia should you have a cesarean delivery (Tables 4–7).

1. Did you take formal prenatal classes?
2. YES/NO
3. What is your age in years? _____
4. Marital Status (circle one):
5. Single
6. Married
7. Cohabitate
8. Divorced
9. Widowed
10. Ethnicity (self-identified; circle one):
11. Caucasian
12. African-American
13. Hispanic
14. Asian/Pacific Islander
15. Native American
16. Other (specify): ________________________
17. How many years of formal schooling have you completed (circle one)?
18. < 12 Years
19. High School
20. Some College
21. Bachelor's Degree
22. Graduate Degree
23. What is your approximate yearly household income (add everyone in the household)?
24. Less than $20,000
25. $20,001 to $50,000
26. $50,001 to $100,000
27. $100,001 to $200,000
28. More than $200,000
29. Are you a health care professional (for example, a physician, nurse, physical therapist)?
30. YES/NO
31. As an adult, have you had previous surgery requiring anesthesia?
32. YES/NO
33. If your answer to #8 was YES, list the type of surgery and type of anesthesia for each operation, and the number of cesareans and type of anesthesia. Also, circle any unpleasant side effects you may have had.
34. During your cesarean would you prefer to be (circle):
35. Asleep
36. Awake
37. Awake but sleepy
38. After surgery do you expect to be in (circle):
39. No pain
40. Some pain
41. Moderate pain
42. Severe pain
43. Indicate on the line below (mark with an X) how much pain you would tolerate rather than expose your baby to any medication you receive:
44. 0 |——————————————————————| 10
45. (No Pain) (Worst pain imaginable)
46. What is your biggest fear with spinal/epidural anesthesia (circle one)?
47. Pain on insertion
48. Failure to provide pain relief.
49. Paralysis
50. Backache
51. Headache
52. Other (specify): ________________________

We want to determine your preferences for each of the following possible outcomes of anesthesia care (i.e. which ones you think are better or worse than the others). Please carefully listen to each of the following descriptions of outcomes you could experience as a result of your anesthesia and surgery. Assume that each situation described is equally likely and that each condition will last an equal length of time. First, rank each of these outcomes in relation to each other from 1 to 10 from the most desirable (1) to the least desirable (10). Next, pretend that you are given $100 in order to prevent the following undesirable outcomes of anesthesia. Distribute the $100 such that the more money you spend on a condition, the less likely that it will occur. Thus, you should spend more money on outcomes you most want to avoid. You must spend all of your $100 (and no more than that). You can choose to allocate $0 to only one of the outcomes described. Please note that many of these outcomes have been made to sound extreme to help you decide your priorities. Most people do not in fact usually experience multiple significant side effects with anesthesia. Treatment for pain or side effects is always available.

When providing pain relief, anesthesia doctors may need to make treatment decisions which “trade-off” relieving pain during and after cesarean section with a possible increased risk of side effects. Look at the table below and choose the pain medication dose (lowest, moderate, or highest) that best describes your preference for pain relief balanced with the possible risk of side effects during and after your surgery and anesthesia. This information will not be used to select the medication used in your current anesthetic.

### (B). Patient Expectations and Preferences Regarding Anesthesia Care Surrounding Cesarean Delivery Postoperative Survey

Subject # _____

The following questions are about the cesarean section you had during this hospitalization.

1. What was more painful, the IV or the SPINAL/EPIDURAL placement (circle)?
2. YES/NO
3. Did you have any unpleasant side effects during or after surgery?
4. YES/NO
5. If your answer to #2 was YES, list what you experienced (circle):
6. Nervous before surgery
7. Nausea
8. Vomiting
9. Pain during surgery
10. Pain within 24 hours after surgery
11. Shivering during surgery
12. Itching within 24 hours after surgery
13. Other (specify): ________________________

We want to determine your preferences for each of the following possible outcomes of anesthesia care (i.e. which ones you think are better or worse than the others). Assume that each situation described is equally likely and that each condition will last an equal length of time. First, rank each of these outcomes in relation to each other from 1 to 10 from the most desirable (1) to the least desirable (10). Next, pretend that you are given $100 in order to prevent the following undesirable outcomes of anesthesia. Distribute the $100 such that the more money you spend on a condition, the less likely that it will occur. Thus, you should spend more money on outcomes you most want to avoid. You must spend all of your $100 (and no more than that). You can choose to allocate $0 to only one of the outcomes described.

Look at the table below and choose the pain medication dose (lowest, moderate, or highest) that best describes your preference for pain relief balanced with the possible risk of side effects during and after your surgery and anesthesia.
